# A Theranostic Cathepsin Activity-Based Probe for Noninvasive Intervention in Cardiovascular Diseases

**DOI:** 10.7150/thno.34402

**Published:** 2019-08-12

**Authors:** Tommy Weiss-Sadan, Yael Ben-Nun, David Maimoun, Emmanuelle Merquiol, Ihab Abd-Elrahman, Israel Gotsman, Galia Blum

**Affiliations:** 1 Institute for Drug Research, School of Pharmacy, Faculty of Medicine, The Hebrew University, Jerusalem, Israel, 9112001.; 2 Heart institute, Hadassah, University Hospital, Jerusalem, Israel 91120.

**Keywords:** cathepsins, macrophages, photodynamic therapy, activity-based probes, atherosclerosis

## Abstract

Despite the common use of lipid-lowering medications, cardiovascular diseases continue to be a significant health concern. Atherosclerosis, one of the most frequent causes of cardiovascular morbidity, involves extensive inflammatory activity and remodeling of the vascular endothelium. This relentless inflammatory condition can ultimately give rise to clinical manifestations, such as ischemic heart disease or stroke. Accumulating evidence over the past decades implicates cysteine protease cathepsins in cardiovascular disorders. In particular, Cathepsins B, L, and S are over-expressed during vascular inflammation, and their activity is associated with impaired clinical outcomes. Here we took advantage of these molecular events to introduce a non-invasive detection and treatment approach to modulate vascular inflammation using a Photosensitizing quenched Activity-Based Probed (PS-qABP) that targets these proteases.

**Methods:** We tested the application of this approach in LDL receptor-deficient mice and used non-invasive imaging and heart cross-section staining to assess the theranostic efficacy of this probe. Moreover, we used fresh human endarterectomy tissues to analyze cathepsin signals on gel, and verified cathepsin identity by mass spectrometry.

**Results:** We showed that our PS-qABP can rapidly accumulate in areas of inflammatory atheromas *in vivo*, and application of light therapy profoundly reduced lesional immune cell content without affecting smooth muscle cell and collagen contents. Lastly, using human tissue samples we provided proof-of-concept for future clinical applications of this technology.

**Conclusions:** Photodynamic therapy guided by cysteine cathepsin activity is an effective approach to reduce vascular inflammation and attenuate atherosclerosis progression. This approach could potentially be applied in clinical settings.

## Introduction

Despite the widespread usage of statins and other lipid-lowering medications, cardiovascular morbidity is still a major health issue 1. A primary cause of cardiovascular diseases is atherosclerosis, a chronic inflammatory condition of the vascular wall that is driven by cholesterol deposition 2. These cholesterol particles trigger the infiltration of immune cells into the endothelial subspace and spark an inflammatory reaction that leads to the development of arterial plaques. While most of these arterial lesions remain clinically "dormant", in the long run, some of them will progress and bring about the clinical manifestations of vascular disease, such as ischemic heart disease and stroke 3.

Over the last decade, numerous efforts have been made to characterize clinically dangerous plaques (vulnerable plaques). In addition to reduced fibrous cap thickness and large necrotic areas 4, these vulnerable lesions exhibit higher activity of proteolytic enzymes 5. Several classes of tissue remodeling proteases were characterized over the past few years and include the matrix metalloproteases and serine hydrolases 5. Moreover, evidence from our lab and others have established that the cysteine proteases cathepsins B and S (hereafter abbreviated as CTSs) are key tissue remodeling factors that are typically expressed in advanced arterial lesions 6, 7. Therefore, these molecular signals were explored for their clinical utility 8, 9 and adapted for more advanced imaging modalities such as PET/CT 10 to conceptualize the clinical application and early identification of vulnerable plaques.

An extension of this approach is to take advantage of these molecular cues and deliver a compound that could ameliorate the inflammatory activity of these vulnerable plaques. Such a therapeutic modality has been successfully demonstrated to alleviate cancer progression 11. This method is capitalized on the backbone of our previously reported CTSs inhibitor GB137 12. This qABP covalently modifies the active CTS-proteases and contains three main features: (a) a short recognition peptide for CTSs. (b) An electrophile warhead, acyloxymethyl ketone that forms an irreversible covalent bond with the active site cysteine residue and (c) a reporter element that remains silent until the protease removes the quencher. In our current design (i.e., YBN14), we replaced the fluorescence reporter system with a photosensitizing element that can generate a fluorescent signal when bound to CTSs and when illuminated by near IR-light produces reactive oxygen species. Thus, our diagnostic qABP, GB137, is transformed hereafter into a theranostic tool that benefits from both diagnostic and therapeutic modalities. The application of this technology has been previously demonstrated in a mouse model of aggressive breast cancer, which appears to have elevated cathepsin levels. In that case, our probe enabled the rapid and selective non-invasive imaging of subcutaneous tumors and safely hampered tumor growth by inducing macrophage apoptosis in response to focal light treatment. Moreover, this effect occurred without any noticeable signs of skin or metabolic toxicities 11. Hence, this approach, dubbed photodynamic therapy (PDT), has an advantage over the standards of protease inhibitors as it can attenuate plaque inflammatory activity through focal light treatment and therefore, reduce the amount of immune cell content in the plaques.

This rationale has prompted the design of chlorine *e6* on a polylysine polymer by Shon SM *et al.,* 13. This polymer aimed at targeting cathepsin B in ApoE knockout mice, to attenuate atherosclerosis burden. Using this method, Shon SM *et al.*, significantly reduced the content of lesional macrophages but at a cost of a highly invasive procedure 13. This fact highlights the need for a different design to minimize this cumbersome procedure. Here we demonstrate the application of our noninvasive PDT approach in the LDL receptor-deficient (*Ldlr^-/-^*) mice using covalent probes. We show that YBN14 accumulates in atherosclerotic lesions of mice and attenuate disease burden by reducing the number of inflammatory cells and potentially increases plaque stability by increasing its collagen content. We also test the clinical application of such therapeutic modality in humans by showing that YBN14 binds to activated CTSs in patients derived tissue specimens.

## Materials and Methods

### Chemical synthesis of photodynamic quenched activity-based probe YBN14

YBN14 theranostic probe was synthesized followed Ben-Nun *et al.*, 11 and is described in the supplementary materials.

### Animals

LDL receptor-deficient mice (*Ldlr^-/-^*) in the background of CL57BL/6 purchased from the Jackson Laboratory (Bar Harbor, ME USA) were housed in specific pathogen-free conditions at the Hebrew University and were used with the approval of the animal ethics committee of the Hebrew University. Females, at the age of eight weeks and same weight, were used for experiments and challenged with a high-fat diet (HFD) (TD88137, Envigo) or normal chow (NC) for 12 weeks.

### Non-invasive *in vivo* imaging and light therapy

Mice were fed high-fat diet (HFD) for 12 weeks and then were injected with YBN14 (75 nmol/mouse, dissolved in 10% DMSO in PBS in a final volume of 100 µL) through the tail vein. For *in vivo* imaging, mice were kept under anesthesia by isoflurane and imaged at different time intervals using the IVIS Kinetic (PerkinElmer, USA) equipped with 710/760 nm excitation/emission filters. For photodynamic therapy, mice were fed HFD or normal chow for twelve weeks; then injected with YBN14 once a week for three weeks in combination with two light treatments (760nm, 50mW, 20min) given 6 h and 12 h post each injection.

### Aortic atherosclerosis lesion analysis

Mice were sacrificed, and the hearts were carefully dissected and embedded in optimal cutting temperature (OCT) tissue compound (Sakura, Tokyo, Japan) and stored at -80 ^o^C. Alternate cryosections of the aortic root (7 μm thick) were generated using microtome (Leica Biosystems) giving a total distance of approximately 100 μm from the annulus. Digital images were captured in Nikon-TL microscope.

### Lipids and collagen quantifications in aortic lesions from mice

For lipid quantification, longitudinal sections were fixed in 4% paraformaldehyde (Electron Microscopy Sciences, 15710) for 15 min at room temperature, washed in PBS x1 and covered with propylene glycol (ACROS ORGANICS, 158720010) for 5 min. Cryosections were then stained with Oil Red O (Sigma-Aldrich, O1391) for 10 min at 37 °C and washed once with 60% 2-propanol and distilled water until the dye was completely removed from a negative control slide (containing no tissue). Lipid quantification was performed as described previously 14. Lipid content was calculated as Oil Red O area / total lesion area and averaged over at least two sections per mouse.

Collagen content was determined in methanol fixed tissue sections using the Picro Sirius Red Stain Kit (Abcam, ab150681). Quantitative determination of collagen content was performed on threshold images using adobe photoshop (Adobe). Collagen content was determined as described for lipid content analysis.

All measurements and evaluations of lipids and collagen content in aortic sections were performed in a blinded fashion.

### Immunofluorescence analysis

For immunofluorescence staining, serial cryostat sections (7 μm) of aortic roots were fixed in cold methanol (-20 °C for 5 min), air-dried, and blocked with CAS-Block™ solution (Thermo-Fischer, 008120). Primary antibodies used in this study were as follows: CD11b from eBioscience (clone M1/70,14-0112-81), alpha-smooth muscle actin from Novus (NB600-531). Slides were mounted with DAPI Fluoromount-G^®^ solution (SouthernBiotech, 0100-20) and digital images were taken in Olympus IX83 microscope with UPLSAPO x60 oil lens. Image quantification was performed in ImageJ 15 and the data are expressed as described for lipid and collagen content.

### Serum lipids profile

After 12 weeks of high-fat diet, blood samples were drawn from individual mice and allowed to clot at room temperature for 20 min. The serum was separated by centrifugation (1000 x *g* for 10 min) and stored at -20 ^o^C until analyzed further. Cholesterol and triglycerides assessment were analyzed by an automated clinical diagnostic platform (COBAS c311, Roche) using the following kits: CHOL2, HDLC3, and TRIGL kits from (Roche) according to the manufacturer's protocol.

### Cathepsin activity in human plaques determined *ex vivo* by in-gel fluorescence using YBN14 or GB137

Cathepsin activity in human atherosclerotic tissue was determined as described previously 6, and was dependent upon the agreement on informed consent. Briefly, fresh human carotid plaques were obtained after endarterectomy surgery and washed several times in PBS. Tissues were then immediately put in liquid nitrogen and kept in -80 °C until they were processed further. To determine cathepsin activity by SDS-PAGE, protein extracts from carotid plaques were incubated with YBN14 or GB137, (indicated concentration) for 2 h in labeling buffer (50 mM sodium acetate pH 5.5, 5 mM MgCl_2_, 4 mM DTT) at 37 ^o^C. The reaction was stopped by the addition of Laemmli sample buffer (10% glycerol, 50 mM Tris HCl, pH 6.8, 3% SDS and 5% β- mercaptoethanol, 0.1% bromophenol blue) and heating at 95 ^o^C for 5 min. Equal amount of proteins (50 μg) were separated on a 12.5% SDS-PAGE, and cathepsin activity was determined by the fluorescent signal as measured in an Odyssey scanner (LI-COR Biosciences) at 800 nm for YBN14, or in a Typhoon scanner for GB137.

### *Ex vivo* imaging of human atherosclerotic lesions

Fresh carotid plaques were obtained as described above, washed thrice with acetate buffer (50 mM sodium acetate pH 5.5, 5 mM MgCl_2_). Intact tissues were immediately labeled with YBN14 (20 µM) for 4 h and then washed with acetate buffer to remove unbound probe. As a negative control, part of the samples was pre-incubated with GB111-NH_2_ (10 µM), a pan cathepsin inhibitor 16, 17, for 1 h. Labeled tissues were then imaged in IVIS Kinetic system (PerkinElmer, USA) equipped with 710/760 nm excitation/emission filters.

### Mass spectrometry identification of cathepsins in human plaque

Identification of cathepsin proteases by SDS PAGE carried out according to Kaschani F *et al.*, 18, with several modifications. Protein extracts (50 μg) from human carotid plaque tissue were labeled with YBN14 (20 µM) for 2h in labeling buffer as described above. The reaction was carried out at 37 ^o^C and stopped by the addition of Laemmli sample buffer in protein denaturing conditions (95 °C) for 5 min. Proteins were separated on 12.5% SDS-PAGE and scanned in an Odyssey scanner (LI-COR Biosciences) at 800 nm. Fluorescent gel printout was used as a template to cut small gel slices corresponding to labeled proteases for subsequent in-gel protein digestion 18. Proteins were reduced and alkylated with 1,4-Dithiothreitol (Sigma-Aldrich, D9779) Iodoacetamide (Sigma- Aldrich, I1149) and digested with mass spectrometry grade trypsin (Pierce, 90058) as described in the manufacturer's protocol. Digested peptides were desalted on C18 Stage-Tips 19. The peptide mixture was injected to 0.75 µm C18 column on a nano-2D HPLC system (Eksigent) coupled to the MS. Mass spectrometry was carried out with Orbitrap (Thermo-Fischer). MS^2^ spectra data were searched using the MASCOT algorithm against human proteome. Mascot searches allowed for oxidation of methionine residues (16 Da), static modification of cysteine residues (57 Da; due to alkylation with iodoacetamide), tryptic peptides with one missed cleavage allowed, and a mass tolerance set to ± 0.25 Da for precursor mass and ±0.35 Da for product ion masses. The 'Decoy Database Search' was turned on. The resulting MS2 spectra matches were assembled and filtered according to MASCOT Protein scores.

### Statistical analysis

Statistical analyses were performed in GraphPad Prism 7. All data assumed to follow a normal distribution and statistical tests are described in details in the figure legends. *P* values lower than 0.05 were considered to be statistically significant.

## Results

### Preclinical detection of atherosclerotic lesions *in vivo* by a quenched photodynamic activity-based probe

**Figure 1:**
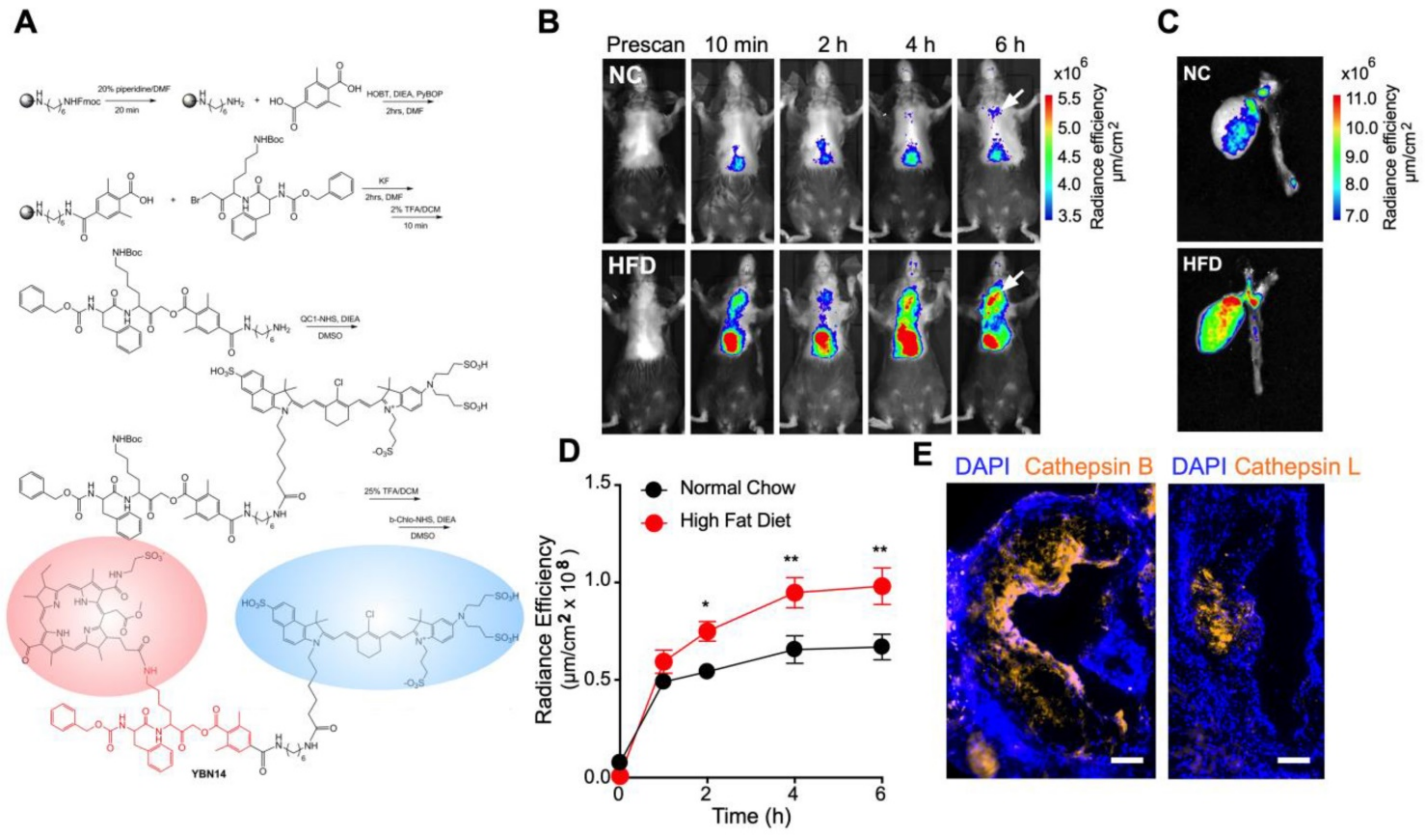
** Kinetic assessment and bio-distribution of YBN14 in atherogenic mice (A)** Schematic description of YBN14 synthesis, for detailed method see Supplementary Method 1. Please note the quencher (blue oval) and the photo-sensitizer (red oval) attached to GB111-NH_2_ scaffold (red). **(B)** *Ldlr^-/-^* mice were fed on normal chow (NC) or HFD for 12 weeks to induce atherosclerosis. After 12 weeks, YBN14 was administered intravenously, and the fluorescent signal from the chest area was captured over time non-invasively by an *in vivo* imaging system (IVIS). YBN14 kinetics and bio-distribution were assessed. Representative image of YBN14 fluorescence signal over time. **(C)** *Ex vivo* images of the hearts and the aortas of corresponding mice. Fluorescent signal intensity is presented as a spectral heatmap, and values represent the radiance efficiency (µm/cm^2^). **(D)** Quantitative analysis of the YBN14 fluorescent signal in NC group (n=5) and HFD group (n=7) corresponding to the region of the heart, indicated by arrows in panel (B). **(E)** Representative longitudinal micrographs for cathepsins B and L expression in aortic lesions from HFD mice (scale bar is 100 µm). Data present the mean ± SEM, and the statistical difference was determined by student's t-test and the False Discovery Rate (FDR) to adjust for multiple comparisons*.* * *P*<0.05, **P<0.01.

Recently we demonstrated the application of ABP to detect cathepsin activity *in vivo* in a diabetic murine model for atherosclerosis 8. Similarly, we set out to assess the efficacy of our PS-qABP, YBN14, (Figure 1A) to accumulate at the regions of the nascent plaques in LDL receptor-deficient mice (*Ldlr*^-/-^) 20. To induce atherogenesis in this animal model, *Ldlr*^-/-^ mice were given high-fat diet (HFD) or normal chow (NC) as a control for twelve weeks. This time frame is sufficient to cause full-blown atherosclerosis in this animal model as evident in our experience and others 21, 22. During this period, mice weight was carefully monitored to make sure they had tolerated this special diet formulation, being 42% of calories coming from fat. After 12 weeks, the HFD group gained 50% of their initial weight values compared to the NC group that gained only 17% (Figure S1). This data suggests that mice on the high-fat diet consumed enough fat to drive the pathologic process of vascular lesion development. To test whether YBN14 can indeed target vascular lesions *in vivo*, we injected YBN14 to the animals and visualized its fluorescent signal using the IVIS platform. Consistent with our previous observations 8, cathepsin activity was increased in mice fed on HFD as evident by the rise in fluorescent signal in the aortic root (Figure 1B, C). Furthermore, quantitative analysis of YBN14 signal suggested it could clearly distinguish between NC and HFD fed animals as quickly as 2 h post injection, and that signal reached a plateau after 4 h (Figure 1D). Moreover, longitudinal sections of the aortic root confirmed the presence of CTS B and CTS L in aortic lesions, with a noticeable predominance of CTS B (Figure 1E). Collectively, this data highlights the utility of cathepsin activity as a biomarker for pathological plaques and provides kinetic information on YBN14 distribution at the lesion area.

### Photodynamic therapy ameliorates atherosclerosis burden *in vivo*

**Figure 2:**
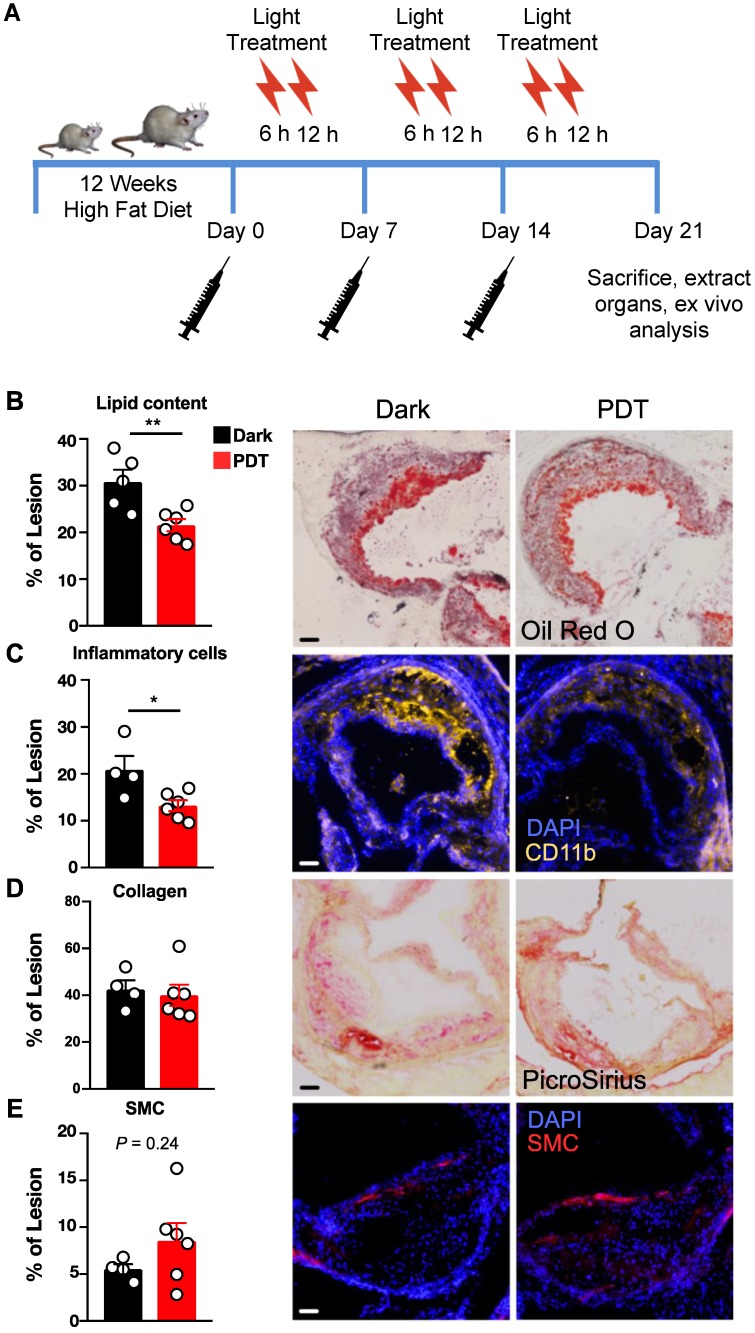
** Cathepsin based phototherapy attenuates vascular inflammation *in vivo*. (A)** Schematic presentation of experimental design. Female *Ldlr*^-/-^ mice were challenged with HFD for 12 weeks and injected with PS-qABP (YBN14) once a week for three weeks with or without light treatment (PDT or Dark respectively). Analysis of lipid content **(B)**, inflammatory cells **(C)**, collagen content **(D)** and smooth muscle cells **(E)** were evaluated in longitudinal cross-sections of the aortic sinus for each experimental group. Representative images are presented on the right panel for each bar graph, at least two sections were analyzed per mouse, and each data point represents the mean value determined for each mouse. * *P*<0.05, **P<0.01, scale bar is 100 µm.

Since YBN14 can efficiently target lesional cathepsins, we next sought to determine its therapeutic efficacy. For this purpose, *Ldlr*^-/-^ mice were given high-fat diet for twelve weeks and received YBN14 followed by two consecutive light treatments to induce photodynamic therapy (PDT). This treatment protocol was given once a week over a time interval of three weeks (Figure 2A). Atherosclerotic lesion burden was assessed in aortic cross sections and analyzed for lipid content, inflammatory markers, collagen content and smooth muscle cells (SMC). Similar to our previously reported observations in a cancer model 11, photodynamic therapy significantly reduced the content of lesional macrophage foam cells as determined by Oil Red O and CD11b stains (Figure 2B, C). Moreover, these changes in plaque composition were not accompanied by a simultaneous reduction in collagen or SMC content (Figure 2D, E) suggesting an overall improvement in plaque stability and inflammatory activity.

### Labeling cathepsin activity in human atherosclerotic plaques

**Figure 3:**
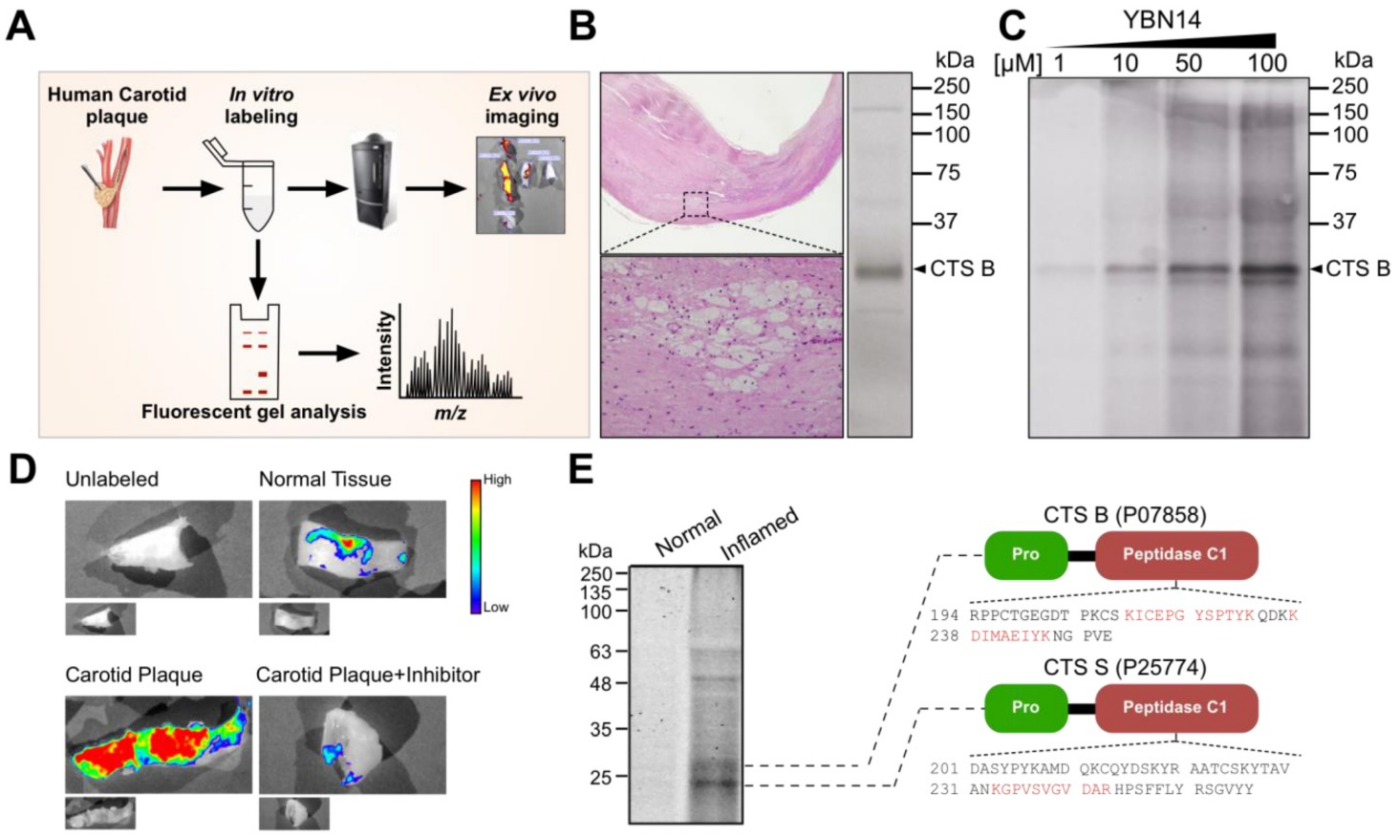
** Labeling cathepsin activity in human atheromatous plaques. (A)** Schematic description of *in vitro* labeling and detection of cathepsin activity in human carotid plaques (CPs). **(B)** Endarterectomy carotid tissues stained with Hematoxylin & Eosin showing foam cells (higher magnification) in micrograph section, left, corresponding tissue labeled with GB137 (1µM) for 2 h, cell lysate was analyzed by fluorescent SDS PAGE, right. **(C)** Dose-response of YBN14 in human CP tissue. Total protein extracts were labeled with increasing concentrations of YBN14 for 2 h, YBN14 labeled cathepsins were visualized by in gel fluorescence. **(D)** Analysis of cathepsin activity in human CP. Fresh CP or adjacent “normal” tissue were labeled with YBN14 for 4 h and then imaged by IVIS. Two samples served as negative controls: (1) CP+ inhibitor sample that was treated with GB111-NH_2_, an established cathepsin inhibitor or (2) unlabeled tissue that was incubated with vehicle. **(E)** Mass spectrometry identification of cathepsins B and S. Fluorescent gel analysis was performed as described in panel B. Thin slices (corresponding to the marked bands) were cut off the gel, digested by trypsin, and analyzed by mass spectrometry. Unique peptides are displayed in red and overlaid on their corresponding region on the protein.

To check the potential application of this emerging technology in humans, we first examined whether cathepsins could bind YBN14 in human atheromas. For this purpose, we took fresh atherosclerotic plaques from patients, labeled them *in vitro* and analyzed cathepsin activity by complementary approaches as summarized in Figure 3A. Briefly, atherosclerotic tissues were labeled with YBN14 for 4 h and cathepsin activity was visualized by SDS-PAGE or by IVIS 12. We started by labeling fresh endarterectomy tissues with GB137 12, that has an analogous structure to YBN14, but only generates a near IR fluorescent signal. As anticipated, CTS B activity was evident on gel (Figure 3B right) and an independent pathological examination confirmed the presence of foam cells in that tissue [(higher magnification) (Figure 3B left)], indicating of a relationship between inflammatory immune cells and cathepsins. Next, we determined the optimal concentration for YBN14 to label endarterectomy carotid tissues. To this particular end, we tested escalating doses of YBN14 in fresh carotid samples and found that YBN14 given at 10-100 µM was sufficient to give a clear signal for CTS B, as predicted by their molecular signature on the gel (Figure 3C). In the next step, we wanted to provide a proof of principle for the diagnostic utility of YBN14 in the clinical settings. We therefore, labeled fresh human carotid plaques (CP) with YBN14 and visualized their fluorescent signal by IVIS. Consistent with our data so far, cathepsin activity is much higher in CP areas compared to nearby semi normal tissue (Figure 3D). Moreover, pretreatment with the pan-cathepsin inhibitor GB111-NH_2_ completely suppressed YBN14 fluorescent signal from cathepsin activity, indicating that YBN14 targets lesional cathepsins (Figure 3D). In a complementary set of experiments, we verified that YBN14 binds to cathepsins by mass spectrometry approach. We adapted an established method for *in situ* characterization of proteases by mass spectrometry 18. Briefly, inflamed vascular region or semi normal adjacent tissue section were labeled with 20 µM YBN14 for 4 h. Then, total cell extracts were resolved on a gel and visualized by a near-infrared scan. Thin fluorescent gel slices were cut out of the gel, and extracted peptides were analyzed by mass spectrometry. In concordance with our data, inflammatory plaque regions displayed higher CTS activity than normal vascular tissues (Figure 3E). Moreover, unbiased mass spectrometry analysis confirmed this activity is attributed to cathepsins B and S as previously described by our group 6. Collectively, these data imply on the potential clinical application of cathepsin based theranostic probes for the detection of inflammatory activity in vascular atheromas.

## Discussion

Cardiovascular diseases remain challenging despite the breadth of lipid-lowering medications. In fact, the inflammatory nature of atherogenesis is a significant contributor to vascular remodeling and its related medical complications 23. Vascular inflammation is driven by different inflammatory cell types including T-cells, B-cells and a sizable proportion of resident macrophages 2, 24. Several reports from our laboratory and others have demonstrated that macrophage inflammatory activity is often associated with the secretion and activation of the cysteine proteases cathepsins B, L and S 6, 25, 26. This fact highlights the possible application of such molecular information to differentiate plaques that are most likely to cause the clinical symptoms of cardiovascular diseases (vulnerable plaques) and treat them in advance to prevent the manifestations of a sudden heart attack and stroke.

In that sense, a concentrated effort to enhance the utility of molecular probes for clinical applications has been made for different types of inflammatory diseases including cancer 11, 27, pulmonary fibrosis 28 and cardiovascular imaging 29. While these probes were indefinitely efficacious in illuminating inflammatory activity, they all lack the inevitable need for a therapeutic application. Hence, in the present study, we introduce a different approach that takes advantage of this technology and improves it by adding a photosensitive element that allows the depletion of inflammatory cells by focal illumination of near- infrared (IR) light.

First, we demonstrate that the PS-qABP, YBN14 can rapidly home in on atherosclerotic lesions *in vivo* using the conventional animal model of *Ldlr^-/-^*mice 20-22. Similar to our previous observations in a diabetic model of atherosclerosis 8, YBN14 rapidly accumulated in the aortic root, where most atherosclerotic plaques build up. This fact, highlights the general application of ABP for non-invasive cardiovascular imaging across different models and regardless of the presence of diabetes that hinders the use of ^18^FDG in the clinical settings to capture inflammatory activity. More significantly, this study demonstrates the potential application of non- invasive phototherapy to treat vulnerable plaques that display a high degree of inflammatory activity. Specifically, the PS-qABP, YBN14 in combination with focal IR light therapy reduced lesional inflammatory cells, without showing any other adverse effect to SMC or the amount of plaque collagen content. Altogether, this data suggest that molecular phototherapy can overall improve plaque stability.

In light of these encouraging results in the murine model, we sought to provide a proof of concept for the application of this technology in the clinical settings. For this purpose, we used fresh CP tissues from patients and labeled them with YBN14. Indeed, those plaques displayed high protease activity at the sites of injury. Moreover, we could validate that YBN14 targets cysteine cathepsins B and S by mass spectrometry, which is consistent with our previously reported observations on these proteases being highly reactive in symptomatic patients 6.

This study together with the data reported by Shon SM *et al.,* 13 demonstrate the potential utility of molecular phototherapy to ameliorate atherosclerosis burden. The advantage of this study is the introduction of a non-invasive "theranostic" approach that combines imaging and therapeutic modalities. Although this technology introduces several challenges before it completely matures into the clinical settings, yet this study consolidates the basis for this molecular entity to be modified in a way to suit the clinical settings. Such a technology has been successfully implemented in our laboratory using gold nanoparticles in a model of breast cancer 27.

## Supplementary Material

Supplementary figures and methods.

## Abbreviations

CP: Carotid Plaque
CTS: Cathepsin
FDR: False Discovery Rate
HFD: High Fat Diet
IVIS: *In Vivo* Imaging System
Ldlr^-/-^: Ldl Receptor Deficient
NC: Normal Chow
PDT: Photodynamic Therapy
PS- qABP: Photosensitizing Quenched Activity-Based Probe
SMC: Smooth Muscle Cells
