# Supplementary material for: A Theranostic Cathepsin Activity-Based Probe for Noninvasive Intervention in Cardiovascular Diseases
Source: Theranostics. 2019 Aug 12;9(20):5731–8. doi: 10.7150/thno.34402 (PMC6735363; doi:10.7150/thno.34402)
Supplement: Supplementary file 1 — Supplementary figures and methods. [file thnov09p5731s1.pdf]

Supplemental information:

Theranostic Cathepsin Activity-Based Probe for Noninvasive Intervention in Cardiovascular Diseases

Tommy Weiss-Sadan[1,#], Yael Ben-Nun[1,#], David Maimoun[2], Emmanuelle Merquiol[1], Ihab Abd-Elrahman[1], Israel Gotsman[2]*, Galia Blum[1]*

[1]*Institute of Drug Research, School of Pharmacy, Faculty of Medicine, The Hebrew University, Jerusalem, Israel, 9112001.* [2]*Heart institute, Hadassah, University Hospital, Jerusalem, Israel 91120.*

[#] These authors contributed equally to this work.

*Corresponding authors: Galia Blum[1], galiabl@ekmd.huji.ac.il and Israel Gotsman[2], igotsman@bezeqint.net

Running title: Theranostic application for qPD-ABP in cardiovascular diseases

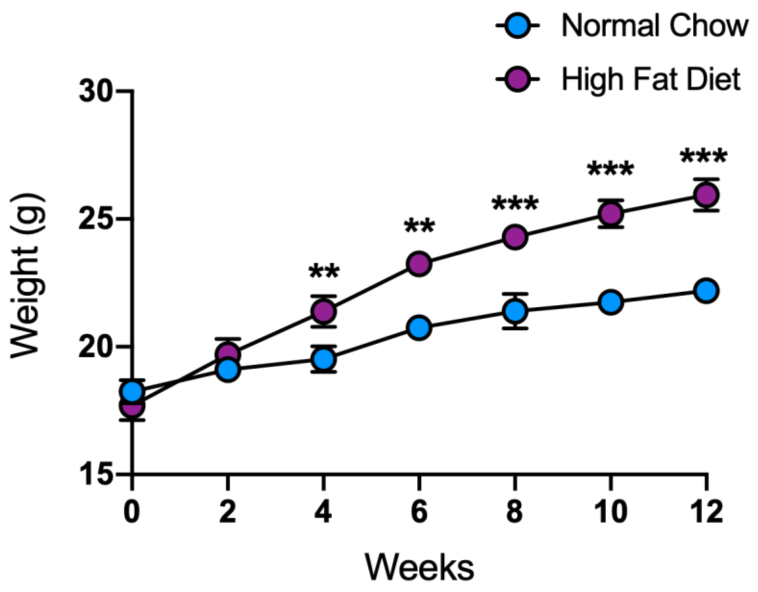

Supplementary Figure 1. Mice weight gain after twelve weeks on a high-fat diet compared to normal chow. Mice were fed high-fat diet (n=7) or normal chow (n=5) for twelve weeks, and their weight was recorded on every other week. Data present the mean ± SEM. Student's t-test used to evaluate the statistical difference between groups and adjusted for multiple hypothesis correction with the False Discovery Rate (FDR). *P* values below 0.05 were considered statistically different. ** $P<0.01$, ***$P<0.001$.

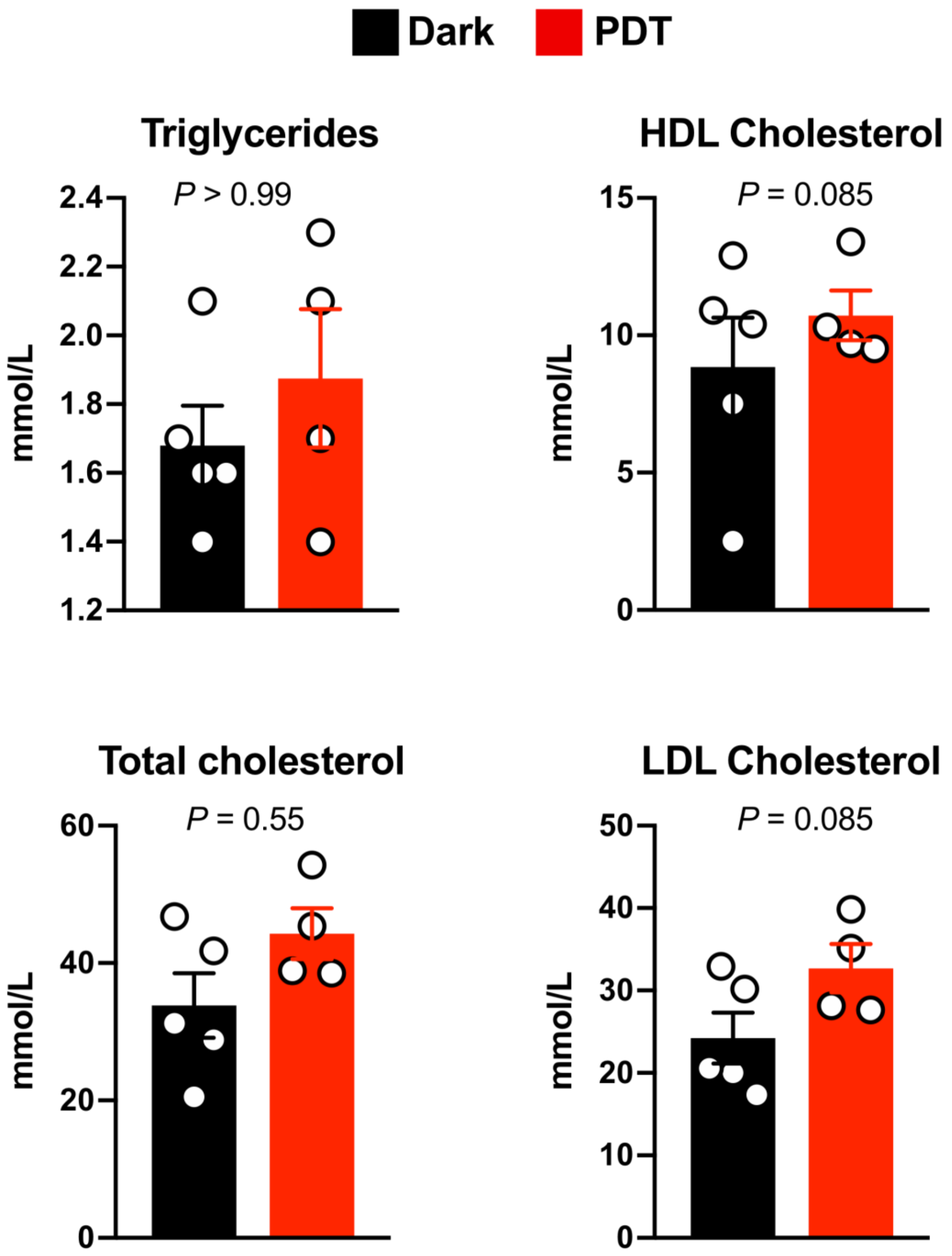

Supplementary Figure 2. Lipid profiles of mice on a high-fat diet. Serum lipid profile of mice fed high-fat diet for twelve weeks. Metabolic measurements were performed in a double-blind manner by external clinical laboratory service. Data summarized in bar graphs present the mean ± SEM of four to five mice per group. The statistical difference for each parameter was determined by a student's t-test and corrected for multiple hypotheses using the false discovery rate (FDR). Therefore, the *P* values represent the adjusted values after FDR correction. There was no significant difference in the metabolic parameters between the PDT and Dark treatment. *P* values are indicated in the figure.

Supplementary method. Synthesis of photosensetizer quenched activity-based theranostic agent Schematic description of YBN14 synthesis and essential elements as previously described [11]. YBN14 is designed according to the principles of GB137, a quenched activity-based probe presented by Blum G and colleagues [12]. The photosensitizer (b-Chlo, Pink) and quencher (QC-1, blue) pair are highlighted. Fmoc, 9-fluorenylmethylcarbonyl; DMF, dimethylformamide; HOBT, 1-hydroxybenzotriazole; DIEA, diisopropylethylamine; PyBOP, (benzotriazole-1-yl-oxy)tris(pyrrolidino)phosphonium hexafluorophosphate; DCM, dichloromethane; Boc, t-butyloxycarbonyl; b-Chlo, Bacteriochlorin (Pink); TFA, trifluoroacetic acid; DMSO, dimethyl sulfoxide.
